# Quality of Life after an Episode of Severe Maternal Morbidity: Evidence from a Cohort Study in Brazil

**DOI:** 10.1155/2018/9348647

**Published:** 2018-07-17

**Authors:** Carina R. Angelini, Rodolfo C. Pacagnella, Mary A. Parpinelli, Carla Silveira, Carla B. Andreucci, Elton C. Ferreira, Juliana P. Santos, Dulce M. Zanardi, Renato T. Souza, Maria H. Sousa, Jose G. Cecatti

**Affiliations:** ^1^Department of Obstetrics and Gynecology, School of Medical Sciences, State University of Campinas, Brazil; ^2^Department of Medicine, Federal University of Sao Carlos, Brazil; ^3^Department of Public Health, Jundiaí School of Medicine, Jundiaí, SP, Brazil

## Abstract

**Objective:**

To assess quality of life (QOL) in women who experienced a severe maternal morbidity (SMM) event and associated factors, in comparison to those who did not.

**Study Design:**

Retrospective cohort study performed at the maternity of the University of Campinas in Brazil, including 801 women with or without SMM, within 6 months to 5 years after delivery. Women were interviewed by phone and data were electronically stored, using the Brazilian version of the SF36 to assess women's self-perception of quality of life. To analyze a possible relationship between SMM and perceived impairment in quality of life, *χ*^2^ and Fisher's Exact tests were used. Multiple analysis using Generalized Linear Models was applied to identify factors independently associated with the general health score. The main outcome measures were general and domain-specific SF36 scores on quality of life.

**Results:**

Maternal morbidity conditions were associated with lower scores of patient perceptions of quality of life in the following domains: physical functioning, role-limiting physical, pain, and general health status. A lower level of school education, not having a partner, caesarean section, and history of previous clinical conditions were associated with a worse perception of general health and quality of life.

**Conclusion:**

Health professionals should know the association between life conditions, previous chronic health conditions, and SMM for women during prenatal care to beyond 42 weeks postpartum. Longitudinal and interdisciplinary actions should be put into practice to provide healthcare for these women, with special emphasis on the effective reduction in health inequities.

## 1. Introduction

Recently, there has been a considerable reduction in maternal mortality in Brazil. However, this decrease was not sufficient to reach the Millennium Development Goal (MDG) in 2015. The reduction in maternal deaths is partly explained by more well qualified obstetric care for women in maternity hospitals and emergency facilities [1]. Surviving a potentially life-threatening condition during or after pregnancy and/or childbirth, along with maternal mortality, should also be considered an indicator of quality of obstetric care. Nevertheless, several health and life aspects of these surviving women are still unknown. A follow-up of these women may be required. This is a challenging task, since little is known about the long-term repercussions of complications on the lives of postpartum women [2, 3]. Such knowledge may improve healthcare and prevent further damage to women experiencing such a condition [4]. Ignoring possible long-term repercussions after exposure to a life-threatening condition may hinder the desirable convergence between a reduction in maternal deaths and a decrease in severe pregnancy-related complications [5].

The combination of pregnancy and severe life-threatening complications may trigger intense physical and psychological distress, which can culminate in posttraumatic stress disorder (PTSD), among other adverse consequences for these women [6, 7]. Survivors of obstetric complications are more physically and socially vulnerable. These women are more prone to develop postpartum mental issues, such as postpartum depression, anxiety, and sexual disorders [8–11]. Six months after delivery, women who experienced severe bleeding, severe preeclampsia (including HELLP syndrome), sepsis, or uterine rupture had a worse evaluation of their own overall health status [9].

Adverse consequences are not limited to the postpartum period. Women who particularly suffered from severe maternal morbidity may have long-term negative consequences after experiencing such episodes. Consequences may affect specially women who underwent operative delivery [12, 13]. Some authors reported that survivors of life-threatening conditions have described their fear of death, loss of hope, concerns about possible upcoming surgical procedures, memory lapses, mourning for the loss of their babies or their reproductive capacity after hysterectomy, feelings of loss of female identity, among others [5, 14]. Therefore, it is necessary to comprehensively understand how severe maternal morbidity influences women's global perception of their quality of life (QOL) after such events. The main purpose of this study was to assess the occurrence and factors associated with the perception of impaired quality of life among women who experienced a severe maternal morbidity event, in comparison to those who did not.

## 2. Methods

This study is part of the cohort study “Multidimensional assessment of the impact of severe maternal morbidity on women's health and life”, carried out at the maternity hospital of the University of Campinas in Brazil [2, 15]. Severe Maternal Morbidity episodes were considered as exposure for women discharged from the ICU. Women who had been exposed to those events were included in the “SMM group”, following the WHO concept and criteria [16]. The nonexposed group comprised women who had a low-risk term delivery at the same facility. These women had given birth to live, healthy children after 37 weeks of gestation or more. Each control was randomly selected according to time of delivery that was close to the year of delivery of each SMM case, in order to keep a balance of time of delivery between groups. The recruitment period ranged from January 1^st^, 2008, to December 31^st^, 2012.

Data were collected from June 2012 to July 2013, including women who had delivered at least 6 months before the first interview. The period between the first interview and childbirth ranged from 6 months to 5 years; however it was determined to be similar in both groups and was considered in the analysis. Sample size for evaluation of quality of life was defined from previous studies [17, 18]. Accordingly, 50% of women had serious physical and emotional problems during the first year after delivery. Assuming an absolute difference of 11% between both groups, with a type I error of 5% and a type II error of 10%, each group would require 337 women, in a total of 674.

From ICU and hospital records, 1,157 women matched selection criteria for both groups. Of the total number, 840 women were successfully traced by phone. Women were then invited to participate in the study. Those who agreed were immediately interviewed by telephone after recording their consent, or another phone call was made to carry out the interview. Aspects evaluated at that time were perception of health-related quality of life through the SF36 questionnaire, and posttraumatic stress disorder and further information on both reproductive and general health were obtained from medical charts.

Quality of life was assessed through the Brazilian version of the Medical Outcomes Study 36–Item Short-Form Health Survey, the SF36 [19, 20]. The questionnaire was applied at one point in time during a telephone interview by trained interviewers and the mean time for telephone interview was 15 minutes. Two interviewers were trained for interviews using tele-research and five were trained for face-to-face interview. The training was carried out by researchers with expertise in tele-research. There were two days of training with practical activities of telephone interview and face-to-face interview. Questions and answers fed an online questionnaire, which was also digitally recorded. This procedure is described as Computer-Assisted Telephone Interview (CATI), a feasible tool to obtain health information [21], including research on quality of life using the SF36 [22]. A research supervisor listened to a random sample of around 5% of recorded interviews to check for consistency of information stored in the digital database, in an attempt to promote healthcare quality.

The SF36 is a generic instrument for multidimensional evaluation of quality of life developed from the Medical Outcomes Study [23]. It was validated for the Portuguese language and the instrument has already been applied at some Brazilian studies [19, 24, 25]. The questionnaire has also been used for general evaluation of health-related quality of life in women within 6 to 12 months after severe maternal morbidity events [9, 26–30]. No other instrument was used for evaluating the quality of life, only the SF-36 as originally planned [2]. In fact, there is no other specific instrument for pregnancy already available. WHO is now doing an effort to reach such an instrument that could be used for this purpose during and after pregnancy, but it is not yet available. We supported our choice in other studies that used the SF-36 with women during pregnancy, who experienced obstetric and postpartum complications [9, 26–30].

The questionnaire is easily applied and contains 36 items divided into eight domains. Two summarized components (physical health and mental health) are derived from these 8 domains. Domains of functional capacity (domain 1), physical aspects (domain 2), and pain (domain 3) are correlated with the physical component. Domains of general health status (domain 4), vitality (domain 5), and social aspects (domain 6) are correlated with physical and mental components. Finally, emotional aspects (domain 7) and mental health (domain 8) are correlated with the mental component [20]. The domain responses are summed to form a score. Higher scores represent a better health status. There is no normal pattern result or cut-off point for the total score. A comparative analysis between two or more groups should thus always be made. The final score ranges from 0 to 100. The latter score corresponds to the best perception of quality of life.

Data were collected using an online platform (Lime Survey®) and analyzed with SPSS® version 23 (IBM, Armonk, NY, USA). After completion of data collection, an intense and detailed process of data management for consistency checking was performed, following a routine planned to explore the relationships between several variables recorded. Inconsistencies were corrected whenever identified, using the original forms, clinical records, or recorded interviews or even by phoning women again to ask them about any missing data. For statistical analysis, initially sociodemographic characteristics and information from pregnancy and childbirth were compared between groups of women with or without SMM using Pearson's Chi-square and Fisher's Exact tests. The median and mean values (±SD) of each SF36 domain scores for participants in both groups were assessed using Student's* t*-test. In domain 4 related to general health status, the mean scores were estimated for categories of each maternal or delivery characteristic, using the nonparametric Mann-Whitney* U* test. Finally, multiple regression analysis was performed using a Generalized Linear Model to identify variables independently associated with general health scores, controlled by predictor variables significantly associated with the outcome. The study was approved by the local Institutional Review Board (letter of approval CEP 233/2009). All participants recorded an audio consent form and/or signed a written version of the consent.

## 3. Results

**Figure 1:**
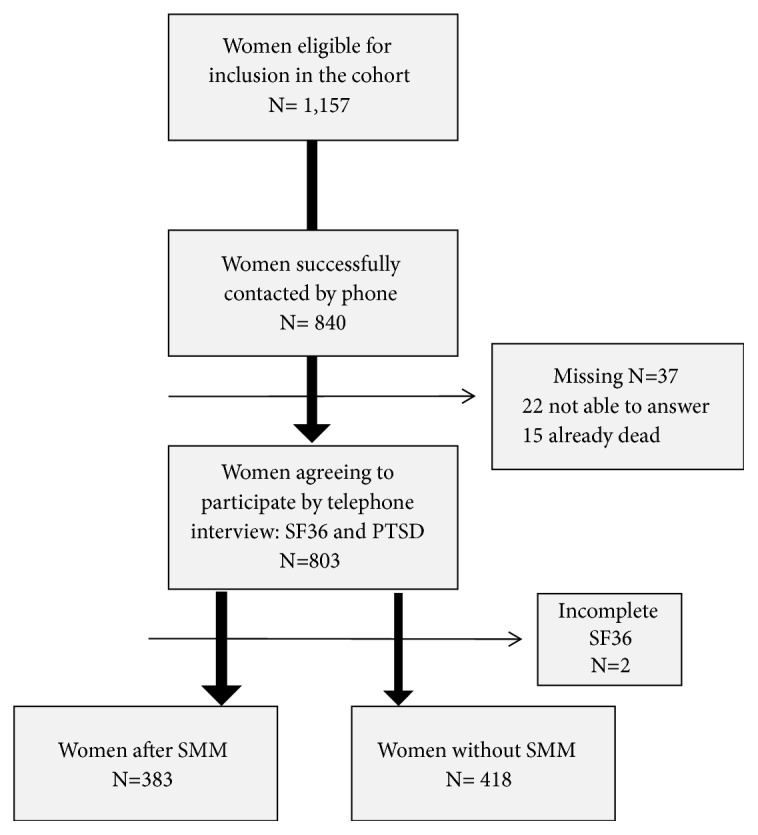
Flow chart of women in the study.

A total of 1,157 women were eligible for the study. Of the total sum, 840 women were traced by phone. However, 37 of these women failed to be interviewed and then 803 answered the SF36 questionnaire. Two of these women did not complete the questionnaire due to personal reasons (a 72.60% trace rate and a 69.23% response rate). Of the total of women who completed the questionnaire, 383 women experienced an SMM episode and were considered exposed patients, while 418 had uncomplicated pregnancies. Among the 37 missing cases, 22 women had been unable to answer the questionnaire for whatever reason and 15 had died (9 from late maternal causes) (Figure 1).

There were significant differences between groups regarding age, mode of delivery, and mainly previous exposure to pathological conditions and smoking (Table 1). Women who had SMM conditions were mostly over 30 years of age, had undergone caesarean section, and had clinical complications, such as hypertensive disorders, obesity, and cardiac disease (65%). Besides higher maternal age, no other life style factors were identified as associated with severe maternal morbidity.

Table 2 shows median and mean SF36 domain scores, according to maternal morbidity. There were significant differences between groups in domain 1 (physical functioning), domain 2 (role-limiting physical), domain 3 (pain), and domain 4 (general health). The mean score in general health domain was 67.2 for women without morbidity, while it was 59.0 for those with severe maternal morbidity. Mean scores in the general health domain were significantly higher in women who had a higher level of school education, had a life partner, and delivered by the vaginal route for the index pregnancy (Table 3). There were no differences between both groups in terms of age, parity, ethnicity, infant gender, or outcome. There was no difference in the SF-36 scores according the time since delivery.

Multiple regression analysis showed that SMM alone was not independently associated with perceived quality of life (Table 4). On the other hand, increasing maternal ages, delivery by the vaginal route, and higher schooling were independently associated with higher general health scores, while having hypertension or some previous clinical conditions (respiratory diseases, thyroid disorders, or HIV) correlated with lower general health status scores.

## 4. Discussion

Quality of life assessment after childbirth showed that women with SMM episodes were older, underwent more caesarean sections, had more morbid conditions prior to pregnancy, and had significantly lower mean scores in SF36 domains 1 to 4, most specifically related to physical aspects and general health, than women without maternal complications. These scores were lower in women with less school education, no partner, who had caesarean section, and with any previous morbid conditions. Conditions that were independently associated with a low score in SF36 domain 4 were hypertension, caesarean delivery, maternal age, respiratory disease, low schooling, and other previous morbid conditions.

This study has some limitations. It was retrospective with SF36 instrument administered only once during postpartum period. Therefore, general health perception before interview was unknown and the time between index pregnancy and interview may have generated recall biases. The SF36 is a generic assessment without clinical aspects and possible risk factors and thus unable to provide a concrete measure of health outcome [20]. Complementary methods using standardized or specific tools, combined with qualitative analyses, are options worthy of exploring. However, the results allow comparisons of QOL scores in women exposed and nonexposed to SMM.

These results are in agreement with other studies investigating what happens to women surviving severe situations during pregnancy, childbirth, or postpartum period [9, 10, 12, 26, 31]. Concern about QOL assessment in women after a SMM event reflects a trend in valuing parameters that are broader than just symptom control, mortality, or life expectancy. It is understood as an individual's perceived position in life in the context of cultural and value systems, related to personal objectives, expectations, health, standards, and concerns [32]. It is a hybrid, biological and social concept that is not exclusively guided by clinical health to assess morbidity outcome [33].

A SMM episode and other severe health conditions, including a traumatic experience during childbirth, are considered to result in adverse effects in these surviving women and their children, family, and society [34–37]. However, scientific management of QOL is challenging. First, there is no consensual definition that can be applied. Second, the concept is linked to a subjective social and cultural burden based on people's self-perception of health status [38]. Recognition and assessment of repercussions of SMM on women's QOL are major steps to demonstrate its importance. This could draw attention to the need for resources also to more common disturbing events experienced by women, even a long period after childbirth [39].

Although there was not a clear association between maternal age and the perception of QOL, adolescent women had a lower mean score in domain 4, probably showing the impact of these new psychological, social, and economic responsibilities with motherhood [40]. Maternal morbid conditions are associated with both increasing and decreasing maternal age. Some studies found a strong association between advanced maternal age and SMM, in addition to higher risks of adverse neonatal outcomes [36, 41]. This is consistent with preexisting medical conditions that are more prevalent in older women.

Results of the physical component analysis (SF36 domains 1 to 4) showed that SMM group had lower scores, indicating that perception of QOL related to physical health may be associated with SMM, as already found elsewhere [10]. Remaining symptoms or “morbidities perceived” by women in the SMM group through their own criteria of severity, discomfort, or interference in daily living routine may have influenced the results [42]. Although this is not the scope of this current analysis, we identified 15 women who had died, nine of them from late maternal causes. We could assume that, if interviewed, they would probably have reported impairment in their QOL.

The SF36 general health component also had lower scores in women with SMM, as already similarly found [9]. Although general health status is correlated with the mental and physical components in SF36 [20], it is worth highlighting that general health status may have been influenced by the physical component, since mental health components showed no differences between groups. We believe that there is a complex interaction between physiological, psychological, and social factors, in addition to a subjective dimension in the disease process. Therefore, even though no significant differences were found in the perception of women's QOL in vitality, social aspects, emotional aspects, and mental health, it is not possible to separate mental and physical health.

Another factor that may have limited the influence of mental health in the global health score was the time elapsed from the event to the interview. Although we did not find differences in QOL when stratifying for time since delivery, longer time span may have favored more adaptive emotional and social responses of women, favoring the development of coping strategies and resulting in a more adapted emotional status.

Women with higher school education and with partners scored higher in the perception of general health status. This corroborates the concept that higher schooling is associated with a healthier lifestyle and access to healthcare and that a partner may offer social and financial support [43, 44]. Chronic conditions correlate with a worse perception of QOL, as our and other studies found for diabetes, hypertension, respiratory disease, HIV, and smoking [29, 45, 46].

The caesarean section was consistently associated with a worse score in domain 4 (QOL related to general health), drawing attention to the extent of postnatal morbidity after caesarean section. Similar results with worse SF36 scores for caesarean section were already reported before [29, 30].

From the results of this study, we can infer that women with severe maternal morbidity episodes, especially if life-threatening and coexistent with some chronic medical condition, who underwent caesarean section and were at the extremes of reproductive age, are at an increased risk of having a worse perception of quality of life, although some recent studies were not able to confirm such findings [47]. Particularly under these conditions, these women need special care. Actions are required to approach the complex interaction between different aspects involved in SMM. Further studies should consider the experience of a severe maternal morbidity as an important impact factor on the quality of life of women. In general, every woman diagnosed as maternal near miss should receive differential care in the postpartum period, either for monitoring clinical repercussions or for identifying difficulties of personal, social, and family reorganization after the experience of such a clinical event. The perception of quality of life does not only depend on the clinical criteria, but also depend on the woman's perception of the impact that an event such as maternal near miss has on her life in a comprehensive way. This screening can be performed in the puerperium by specific instruments such as SF-36 or with specific instruments, as recently proposed by the World Health Organization's maternal morbidity Working Group [48]. Follow-up should include a multiprofessional approach.

## 5. Conclusion

SF36 scores showed significant differences between groups of women exposed to SMM and controls in the domains of functional capacity (domain 1), physical aspects (domain 2), pain (domain 3), and general health status (domain 4). Lower scores in domain 4 were found when women had a lower level of school education, lived without a partner, and had given birth by caesarean section.

Such results may potentially have important implications for clinical practice. Although it is not possible to indicate a clear benefit in favor of any method of delivery, caesarean section was associated with lower scores in general health quality of life and this may have an alarming effect on women's lives, especially in countries that still have high C-section rates such as Brazil.

Even though some aspects of health perception are not necessarily medical or sanitary issues, obstetricians and interdisciplinary healthcare professionals, including primary care workers, must be aware of the potential impact of SMM on women's lives beyond the immediate postpartum period.

## Figures and Tables

**Table 1:** Sociodemographic characteristics of women with and without previous condition of Severe Maternal Morbidity (PLTC+MNM).

**Characteristics**	**SMM Group **		**Group Without Morbidity **		**p-value** ^**∗**^
	**N**	%	**N**	%	
**Age (years)** ^**a**^					**0.003**
<20	16	4.2	24	5.8	
20 -24	58	15.2	81	19.4	
25-29	86	22.5	110	26.4	
30-34	87	22.8	111	26.6	
35-39	82	21.5	56	13.4	
≥40	53	13.9	35	8.4	
**Parity ** ^**b**^					0.307
≤1	98	31.7	110	33.7	
2	93	30.1	103	31.6	
3	55	17.8	65	19.9	
≥4	63	20.4	48	14.7	
**Skin color/Ethnicity ** ^**c**^					0.183
Caucasian	199	52.1	197	47.1	
Non Caucasian	183	47.9	221	52.9	
**Schooling ** ^**d**^					0.257
Up to 4 years	24	6.7	16	4.1	
5 to 8 years	105	29.3	104	26.5	
9 to 11 years (high)	194	54.2	235	59.9	
≥12 years (University)	35	9.8	37	9.4	
**Marital status ** ^**e**^					0.955
With partner	260	82.5	269	83.3	
Without partner	55	17.5	54	16.7	
**Time elapsed between delivery and interview**					0.429
1-2 years	154	40.3	180	43.1	
3-5 years	229	59.7	238	56.9	
**Mode of delivery ** ^**e**^					**<0.001**
Vaginal delivery	67	17.9	217	51.9	
Caesarean section	307	82.1	201	48.1	
**Gender of the child ** ^**∗∗f**^					0.274
Male	204	55.7	214	51.6	
Female	162	44.3	201	48.4	
**Neonatal outcome ** ^**∗∗g**^					0.083
Alive	300	95.8	399	98.3	
Neonatal death	13	4.2	7	1.7	
**Previous maternal condition ** ^**e**^	245	65.2	137	33.1	**<0.001**
**Hypertensive disorders **^**c**^	93	24.3	26	6.2	**<0.001**
**Obesity **^**c**^	77	20.2	52	12.4	**0.004**
**Diabetes **^**c**^	24	6.3	9	2.2	**0.006**
**Smoking **^**c**^	29	7.6	12	2.9	**0.004**
**Cardiac Disease **^**c**^	19	5.0	4	1.0	**0.001**
**Respiratory Disease **^**c**^	19	5.0	5	1.2	**0.003**
**Renal Disease **^**c**^	15	3.9	1	0.2	**0.002**
**Sickle cell/thalassemia **^**c**^	9	2.4	0	0	**0.001**
**HIV **^**c**^	1	0.3	5	1.2	0.220
**Thyroid disease **^**c**^	26	6.8	8	1.9	**0.001**
**Neurological disease **^**c**^	16	4.2	4	1.0	**0.007**
**Collagenoses **^**c**^	7	1.8	5	1.2	0.654
**Neoplasia **^**c**^	5	1.3	4	1.0	0.744
**Others**					
**Total**	**383**		**418**		

MNM: maternal near miss; PLTC: potentially life-threatening condition.

Missing information for a:2; b: 166; c:1; d:51; e: 9; f: 20; g: 82; e:170; e:11 cases.

^*∗*^p value derived from Pearson Chi-square or Fisher Exact tests.

^*∗∗*^Only for pregnancies resulting delivery.

**Table 2:** Median and mean values for SF36 domain scores for groups with and without severe maternal morbidity (means with SD).

**SF-36 Domains **	**SMM Group**				**Group Without Morbidity**				**p-value** ^**∗**^
	**Med**	**Mean**	**SD**	**N **	**Med**	**Mean**	**SD**	**N **	
Domain 1:									
**Physical functioning **	80	75.17	22.38	381	90	83.02	18.26	415	**<0.001**
Domain 2:									
**Role-limiting physical **	100	65.64	40.77	382	100	77.22	35.48	417	**<0.001**
Domain 3:									
**Pain **	61	59.52	24.90	381	62	63.36	22.97	415	**0.018**
Domain 4:									
**General health **	57	59.05	21.07	376	67	67.24	19.63	409	**<0.001**
Domain 5:									
**Vitality **	55	51.81	21.68	381	55	54.52	21.25	411	0.110
Domain 6:									
**Social functioning**	75	67.04	27.28	380	75	70.79	25.69	410	0.063
Domain 7:									
**Role-limiting emotional **	75	58.90	44.47	382	100	63.61	43.01	417	0.127
Domain 8:									
**Mental Health**	56	56.16	21.91	382	60	58.41	22.92	410	0.108

MNM: maternal near miss; PLTC: potentially life-threatening condition.

^*∗*^Student's *t*-test.

**Table 3:** Mean values of SF36 fourth domain scores (general health) according to some maternal and delivery characteristics.

**Characteristics**		**Mean**	**SD**	**n**	**p-value ** ^**∗**^
**Maternal age (y) ** ^**a**^					0.239
≤ 19		57.50	17.01	40	
20-29		63.34	20,69	333	
30-39		64.17	21.31	330	
≥ 40		62.76	19.95	83	
**Number of pregnancies ** ^**b**^					0.394
1		63.09	20.81	204	
≥ 2		61.53	20.94	420	
**Schooling (years) ** ^**c**^					**0.001**
Up to 8		59.38	20.87	242	
Above 8		64.61	20.61	496	
**Ethnicity ** ^**a**^					0.356
White		63.99	20.98	388	
Nonwhite		62.69	20.47	398	
**Marital status ** ^d^					**0.013**
Without a partner		57.81	19.77	103	
With a partner		62.85	20.98	519	
**Time since delivery (y)** ^a^					0.398
< 1		61.26	19.76	111	
1 - <2		63.57	20.95	274	
2 - ≥3		63.74	20.84	401	
**Route of delivery ** ^**e**^					**<0.001**
Vaginal		67.89	20.46	278	
Cesarean section		60.85	20.49	499	
**Child outcome ** ^f^					0.308
Alive		63.55	20.91	685	
Neonatal death		59.20	19.81	20	
**Sex of the child ** ^**g**^					0.440
Male		64.03	20.67	408	
Female		62.64	21.09	358	
**Any morbid condition ** ^**h**^		57.68	20.76	374	**<0.001**
**Hypertensive disorders ** ^**i**^		52.39	19.56	116	**<0.001**
**Obesity ** ^**i**^		58.83	21.26	126	**0.008**
**Diabetes ** ^**i**^		47.84	19.21	32	**<0.001**
**Smoking ** ^**i**^		60.00	18.84	40	0.300
**Cardiac Disease ** ^**i**^		55.09	18.87	23	0.076
**Respiratory Disease ** ^**i**^		48.52	20.47	23	**0.001**
**Renal Disease ** ^**i**^		57.06	22.73	16	0.235
**Sickle cell/thalassemia ** ^**i**^		57.22	26.52	9	0.538
**HIV/AIDS ** ^**i**^		45.50	18.44	6	**0.040**
**Thyroid disease ** ^**i**^		55.79	21.97	33	0.052
**Neurological disease ** ^**i**^		61.10	21.92	20	0.628
**Collagenoses ** ^**i**^		55.67	22.08	12	0.200
**Neoplasia ** ^**i**^		61.50	30.18	8	0.867

^**∗**^Nonparametric test: Mann-Whitney.

Missing information for a: 15 cases; b: 177; c: 63; d: 179; e: 24; f: 96; g:35; h: 26; i:16 cases.

Values in bold mean that they are statistically significant (p<0.05).

**Table 4:** Variables independently associated with mean of general health perception score, domain 4, by multiple regression analysis (Generalized Linear Model) [n=571].

**Variable**	**Coeff.**	**SE coeff.**	**p**
Hypertension	-0.251	0.046	<0.001
Mode of delivery (Vaginal)	0.109	0.030	<0.001
Age (years)	0.007	0.002	0.002
Respiratory diseases	-0.234	0.086	0.007
Schooling (>8 years)	0.082	0.031	0.009
Thyroid diseases	-0.182	0.074	0.014
HIV/AIDS	-0.378	0.170	0.026
Constant	3.861	0.076	<0.001

Coeff.: estimated coefficient; SE coeff.: standard error of estimated coefficient; p: p value.

Independent variables initially considered: group (PLTC, MNM: 1/ Control: 0); age (years); parity (1: 0/ ≥2: 1); skin color/ ethnicity (Caucasian: 0/ Non-Caucasian: 1); schooling (up to 8 years: 0/ >8 years: 1); marital status (without partner: 1/ with partner: 0); time elapsed between delivery and interview (1-2 years: 0/ 3-5: 1); mode of delivery (vaginal: 1/ cesarean: 0); gender of the child (male: 0/ female: 1); Neonatal outcome (alive: 1/ death: 0); previous maternal pathology: hypertension (yes: 1/ no: 0); obesity (yes: 1/ no: 0); diabetes (yes: 1/ no: 0); smoking (yes: 1/ no: 0); cardiac diseases (yes: 1/ no: 0); respiratory diseases (yes: 1/ no: 0); renal diseases (yes: 1/ no: 0); sickle cell/thalassemia (yes: 1/ no: 0); HIV/AIDS (yes: 1/ No: 0); thyroid diseases (yes: 1/ no: 0); neurological diseases (yes: 1/ no: 0); collagenoses (yes: 1/ no: 0); neoplasia (yes: 1/ no: 0).

## Data Availability

The data used to support the findings of this study are available from the corresponding author upon request.

## Abbreviations

CATI: Computer-Assisted Telephone Interview
ICU: Intensive care unit
MDG: Millennium Development Goal
PTSD: Posttraumatic stress disorder
SF36: 36-Item Short-Form Health Survey
SMM: Severe maternal morbidity
QOL: Quality of life
WHO: World Health Organization.
